# Supplementary material for: Characterization of Age-Associated, Neutrophil-to-Lymphocyte Ratio (NLR) and Systemic Immune-Inflammatory Index (SII) as Biomarkers of Inflammation in Geriatric Patients with Cancer Treated with Immune Checkpoint Inhibitors: Impact on Efficacy and Survival
Source: Cancers (Basel). 2023 Oct 19;15(20):5052. doi: 10.3390/cancers15205052 (PMC10605297; doi:10.3390/cancers15205052)
Supplement: Supplementary file 1 [file cancers-15-05052-s001.zip › cancers-2643923-supplementary.pdf]

Supplemental Tables

Table S1. List of contributing centers to the clinical database

Institution	Location
The University of Kansas Cancer Center	Kansas City, KS, U.S.A
Lombardi Comprehensive Cancer Center, Georgetown University	Washington D.C., U.S.A
The University of Texas MD Anderson Cancer Center	Houston, TX, U.S.A
Hammersmith Hospital Campus, Imperial College London	London, U.K.
Vanderbilt-Ingram Cancer Center	Nashville, TN, U.S.A
University Hospital of Parma	Parma, Italy
Azienda Ospedaliero Universitaria Sant'Andrea	Rome, Italy
Istituto Nazionale Tumori IRCSS, Fonfazione Pascale	Napoli, Italy
IRCC Istituto Tumori Giovanni Paolo II,	Bari, Italy
Universita degli Studi di Genova	Genova, Italy
IRCC Ospedale Policlinico, San Martino	San Martino, Italy

Table S2. Baseline inflammatory markers: N: overall sample size; NLR: neutrophil-to-lymphocyte ratio; SII: Systemic Immune-inflammation index.

Pre-treatment marker	N	Overall	< 80 years	≥ 80
**median NLR (range)**	845	3.63 (0.08-43.0)	4.05 (0.08-43)	3.52 (0.23-33.36)
**median SII (range)**	843	848.69 (18.43-19370)	14484 (18.43-19370)	762.07 (37.62-9129.87)
